# Bronchitis in Dogs*Read before the New Jersey State Veterinary Society.

**Published:** 1893-03

**Authors:** R. L. Tucker

**Affiliations:** 59 E. Front Street, Plainfield, N. J.


### BRONCHITIS IN DOGS.*

By R. L. Tucker, D.V.S.

The subject which I propose to bring before you to-day is one which is sometimes met with in the practice of Canine Medicine, which our authors of text books seem to overlook or, in fact, I may say, almost entirely neglect. For it is a sad fact that there is not one text book to-day on Canine Practice that is fit, I may say, to adorn our Veterinary Library. But through careful manipulation and research, I will this afternoon bring before you one of those many diseases known to us in the aforesaid practice, namely, that of *Bronchitis* and its different forms. Its name is almost entirely sufficient to discuss not only its action but the parts affected thereby. The word is derived from two Greek words, viz.: *Bronchæ*, meaning bronchial tubes, and *itis*, an inflammation.

In short, bronchitis is an itis of the mucous membrane, lining the bronchial tubes both large and small When affecting the former it is known as simple bronchitis, or bronchitis proper. When affecting the latter it is known as bronchitis capillaris, or capillary bronchitis. There is also another form of bronchitis, viz.: *Bronchitis Verminosis*, or an affection produced by the germs known as the tæniæ eliptica, which in this last disease are usually found in the part of the small intestine known as the jejunum. But of this we will speak later on.

---

* Read before the New Jersey State Veterinary Society.

*Causes.*—Bronchitis is an affection to which dogs are more or less liable.

It may and does exist as a primary, or secondary disease; in my opinion, more so as the first. When it does exist as the latter, or secondary, it is generally in company with catarrh or any disease affecting the respiratory organs, and we will at once class it under two heads, viz.: Acute and Chronic.

The causes which operate to produce the former are the same as those which tend to cause a continuance of the latter, only they are very much aggravated, viz.: cold, damp atmosphere. The inhalation of obnoxious and irritating gases, neglected or protracted diseases of the respiratory organs, such as catarrh of any of these organs, and of course an extended and prolonged *itis* of the surrounding tissues, and finally the afore-mentioned *bacilli* producing that disease known as *Verminous Bronchitis.*

*Symptoms.*—The symptoms in the dog are very closely allied to those which are met with in the human subject, which, I no doubt, some of you have witnessed for yourselves.

These symptoms, whether well marked or obscure, will entirely depend on the amount of tissue involved. If the malady is only confined to the larger tubes and their branches the breathing will be less disturbed than when the subdivisions are involved, particularly the smaller ones. The cough in the former will also be less frequent, but louder and more sonorous, but with little or no expectoration. This form, however, is rarely witnessed in the canine; if so, it has passed to the more complicated form before the veterinarian arrives, or one is called, as from four to twelve hours is ample time for the tissue to make the change. I shall, therefore, describe the more general symptoms of the acute stage.

The respiration is hurried and labored, the breath may be, and as a rule is, very hot, but I have seen exceptions to this rule more than often showing only the low state of the system; the breath has almost been icy cold, more especially marked a few hours previous to the dissolution; "for do not let us overlook the fact that bronchitis in the dog, more so than in any other animal, is excessively fatal." There is an almost incessant wheezing cough, which finally becomes short and dry, followed by expectoration and vomiting; this last symptom, viz., vomiting, is easily accounted for when we take into consideration the itis, as a rule, will extend from the tubes far up along the trachea and even into the larynx and pharynx. The expectoration, I have marked on several occasions, to be similar to that of pneumonia in its last stages, viz.: that (prune juice) expectoration making me at times doubtful of my diagnosis, which was always verified either by recovery or an autopsy after death. It is usually frothy and not infrequently mixed with blood due to the laceration of one or more small capillaries or arteries in the act of violent coughing or retching. The eyes are very red and the conjunctivæ inflamed, the point of the nose dry and hot, mouth devoid of moisture, tongue parched and showing the appearance of brown fur (another symptom seen sometimes in aggravated cases in man). The pulse is quick and small and the heart's action very jerking. In most of the cases that I have seen the pulse at the pad of the foot was almost imperceptible, while that at the thigh was very plainly felt. I have also been able, in most cases, to get a pulse in one hind foot and not in the other, and in the opposite fore foot to that of the hind where it was felt, showing a very disordered and uneven circulation. On auscultating, the heart emits a thumping noise, but above this can be always distinctly heard the diagnostic mucous rale or rattle of the disease in question. A thin mucous discharge generally sets in at the onset from the nasal organs which, as the disease progresses, becomes copious and muco-purulent and is generally accompanied by violent sneezing, and I have seen cases where this sneezing was so continuous that it would produce nervous prostration, from which the animal would never recover, but die in convulsions. As the malady advances all the above symptoms increase in severity and the final termination is resolution of the parts, which is easily discernible by the diminishing of the fever. The discharge that was heretofore purulent, or muco-purulent, becomes naturally mucous and clear, and loses the pruny smell. The appetite, which was greatly impaired, returns by degrees. The urine that was very dark and scanty becomes lighter and more profuse. The rale ceases to be heard, the pulse resumes natural pulsation, and everything in general proceeds toward recovery; if such is not the case the animal dies either from sheer exhaustion (acute inflammatory fever), or from asphyxia.

The chronic form is usually a sequel of the acute form and more generally met with in animals advanced in years. It very rarely leaves the animal but becomes more or less aggravated, according to the mildness or severity of the changing seasons. The symptoms are invariable, the cough is of a husky nature, brevity of respiration increasing with exertion, expectoration and retching.

This form may and is sometimes confounded with *phthisis*, from which it must be distinguished by the absence of hectic fever, and of the physical signs that are characteristic of the latter, some of which I will herein state, viz.: In bronchitis we are not met with that, "as it is described by some authors," sometimes cough neither do we find, as a rule in bronchitis, the emaciated animal that we have in phthisis. The fœtid or gangrenous breath which we have in phthisis does not accompany bronchitis. Diarrhœas are also present in consumption and not very often seen in bronchitis; and in phthisis the animal gradually passes away, so to speak, without a struggle, while in inflammation of the bronchii suffocation, producing spasms, is the general accompaniment of dissolution In all these cases of chronic bronchitis it is more fully characterized by the formation of solid or tubular concretions of exudative matter within the bronchial tubes, forming, in some instances, to such an extent as to reveal on autopsy polypi in the bronchial structure.

"*Treatment.*"—Immediately symptoms of acute bronchitis are observed, it is advisable to place the animal in a moderately warm, sufficiently ventilated, and dry habitation. With regard to medicinal agents, some recommend from 1–3 grs. tartar emetic in proportion to the size of the patient, and say that it is at the onset very beneficial But in spite of my knowledge of the diseases of the respiratory apparatus, knowing, as you all do, that they are, one and all, depletive diseases, owing to the amount of tissue involved and used up, by the amount of nervous force acted upon, that emetics or aperients, gentle or drastic, are entirely out of place and bad practice, and that a course of medicine entirely stimulative should be resorted to from the onset. Some authors, as Williams, Virchow, &c., recommend aconite as an antiphlogestic agent. Of course I am not here to base my authority against, or on par with their long experience, but simply to say that I have received just as good, and if anything, better results from equal parts of quinine and antipyrin than from aconite. The pulse is, as a rule, quick and weak ; this can be subdued and strengthened by small doses of nitro-glycerine and digitalis alternately, with the regular fever mixture, made up of liquor amnoniæ acetatis, tinct. opii, a small quantity, and some of the alcoholic stimulants; I prefer brandy. If the cough is very hard and dry, a few drops of tinct. of belladonna and acid hydrocyanic will be found beneficial. I have also found that in the cases where sneezing is liable to produce exciting symptoms, doses of morphiæ acetatis, administered hypodermically, will allay it in most cases; if not, why sulphuric æther inhalation will do the work, carried far enough not to produce entire unconsciousness.

As regards to severe emplastrum, such as cantharides, mustard and stimulating applications of turpentine, etc. I think these are entirely out of place, although often well recommended by expert veterinarians on "Canine Practice." Oiled silk and linseed meal poultices are all that are required. I would recommend the former; for by applying poultices, especially when performed by the laity, the sides of the animal are very apt to get chilled in the reapplication of the same. While on the other hand, the oiled silk can be at once applied, and simply an adjustment every two or three days is sufficient to keep it in place. From the susceptibility of the return of the attack, unnecessary exposure to the cold or damp weather should be avoided; and until a thorough restoration to health is established the animal should not be allowed to return to his natural and ordinary life.

*Bronchitis Verminosis.*—I will now endeavor, with your permission, to describe to you a small sketch of the pathology, symptoms and treatment of that other form of bronchitis of which very little is known, viz., that of "*Bronchitis Verminosis.*"

As I stated in the former part of this paper, that its one and only cause was due to a germ peculiar in and to itself. It is known as the "Pentastomum Tanioides."

"*Symptoms.*"—Among the initial symptoms, loss of appetite and disinclination to exercise, together with unsteadiness of gait, amounting in some cases to a sub-paralytic condition of the hinder extremities were the most evident. In almost half of the cases convulsions occur. There is rarely diarrhœa or any other symptom referable to gastro-intestinal disorder, although a large quantity of the parasite is found along the intestinal canal. Cough is not a prominent symptom by any means, it being entirely absent in the majority of cases. When present it is short and husky, very much unlike the regular distemper or bronchial cough. The pulse and respiratory act are very much increased, the temperature elevated to 100½ to 102 degrees F. Whenever food is taken it is, as a rule, vomited. Death takes place in most instances quietly, though sometimes during a convulsion, and as a rule, the pups which have the most convulsions hold out the longest. The duration of the disease is from 3 to 7 or 10 days; and in an epizootic, some months back, in Montreal it lasted about 7 weeks. In inquiring into those cases I found that all, with the exception of 3 or 4, were under 8 months old, and only one of the older dogs died, and it was supposed that this death was due to accident. The hygienic surroundings of these kennels were not at all good. The disease showed itself during a very cold spell; indeed for the first 3 weeks, so I was informed, the weather was below zero. Post mortem examinations were held on several, and in all of them the same basis was found, viz.—this was a fox-hound bitch—on opening the thorax, the lungs were seen to only partially collapse the lower borders of the lobes were firm to the touch and very dark in color. The vessels in the lower mediastinum looked full, and the tissues along that region were extravasated with blood. The auricles, ventricals and pericardium were found to be normal.

After the removal of the lungs there was, upon insertion, the escape of a dirty brown, frothy fluid, through the trachea. The anterior and the middle lobes, the anterior half of the posterior lobes were completely solidified, and were of dark brown reddish color, and contrasted strongly with the normal parts. The pleural surfaces were smooth, with no signs of an exudation.

On section, the lung tissue was of a dark red color, the surface of the section finely granular, and bathed with a quantity of brown serum. The air cells were found to be filled with a solid exudation, and the lungs would fail to inflate when this was attempted, and between the healthy and diseased parts there was found a zone of intense hyperæmia. On slitting up the trachea, small swellings here and there, and within them were found bunches, so to speak, of small parasites. These are most abundant just at the bifurcation of the trachea and tubes, forming a mass 3 to 5 lines in height. The bronchial glands are swollen and enlarged. The stomach is generally filled with a dirty looking fluid. The spleen is normal, jejunum and ileum infested with the tænia. Liver firm and hard, and without an exception a slot in the portal vein. These are, as near as possible, the post-mortem appearance as I could gather from the information that I obtained from my correspondence with one of the professors of the Montreal Veterinary School.

The symptoms, as described by Prof. Conlin, are as follows: The animal is subject to convulsion, during which the animal is violently agitated, stops short, hits itself on the head, rolls over, rubs its nose on the ground, and the jaws are convulsively champed. It devours everything within reach, such as wood, straw, etc., discharges a large quantity of saliva, passes urine involuntarily, and sneezes without intermission. Death, as a rule, ensues. The mucus vent of the nose is found to be red ecchymosis, thickened and ulcerated, the sinuses more or less filled with pus, and in some cases the ethmoid bone has become carious.

The treatment is very unsatisfactory, simply the inhalation of irritating gases, such as super-chlorine, sulphur, etc.

59 E. Front Street, Plainfield, N. J.

